# Major pancreatic resections: normal postoperative findings and complications

**DOI:** 10.1007/s13244-018-0595-4

**Published:** 2018-02-15

**Authors:** Marco Chincarini, Giulia A. Zamboni, Roberto Pozzi Mucelli

**Affiliations:** Istituto di Radiologia, DAI Patologia e Diagnostica, Verona, Italy

**Keywords:** Pancreas, Pancreatectomy, Pancreaticojejunostomy, Pancreaticoduodenectomy, Postoperative complications

## Abstract

**Objectives:**

(1) To illustrate and describe the main types of pancreatic surgery; (2) to discuss the normal findings after pancreatic surgery; (3) to review the main complications and their radiological findings.

**Background:**

Despite the decreased postoperative mortality, morbidity still remains high resulting in longer hospitalisations and greater costs. Imaging findings following major pancreatic resections can be broadly divided into “normal postoperative alterations” and real complications. The former should regress within a few months whereas complications may be life-threatening and should be promptly identified and treated.

**Imaging findings:**

CT is the most effective postoperative imaging technique. MRI and fluoroscopy are used less often and only in specific cases such as assessing the gastro-intestinal function or the biliary tree. The most common normal postoperative findings are pneumobilia, perivascular cuffing, fluid collections, lymphadenopathy, acute anastomotic oedema and stranding of the peri-pancreatic/mesenteric fat. Imaging depicts the anastomoses and the new postoperative anatomy. It can also demonstrate early and late complications: pancreatic fistula, haemorrhage, delayed gastric emptying, hepatic infarction, acute pancreatitis of the remnant, porto-mesenteric thrombosis, abscess, biliary anastomotic leaks, anastomotic stenosis and local recurrence.

**Conclusions:**

Radiologists should be aware of surgical procedures, postoperative anatomy and normal postoperative imaging findings to better detect complications and recurrent disease.

**Teaching Points:**

• *Morbidity after pancreatic resections is high.*

• *CT is the most effective postoperative imaging technique*.

• *Imaging depicts the anastomoses and the new postoperative anatomy*.

• *Pancreatic fistula is the most common complication after partial pancreatic resection*.

## Introduction

The morbidity and mortality of pancreatic surgery have decreased in the last decades thanks to improvements in both surgical technique and postoperative intensive care; the mortality rate originally described in the 1940 series by Whipple was about 25% [1], whereas nowadays mortality is lower than 1% in high-volume centres [2, 3].

Despite this improvement, morbidity still remains high, resulting in longer hospitalisations and greater hospital costs [4–8]. The most common complications following pancreatic surgery are pancreatic fistula (pancreatic fistula), haemorrhage, pancreatitis, porto-mesenteric venous thrombosis, delayed gastric emptying and anastomotic strictures. Among these, pancreatic fistula and delayed gastric emptying represent the most frequent complications [7, 8].

Imaging is not only essential in the preoperative assessment of these patients, but also plays a fundamental role in the postoperative setting to evaluate the presence of complications.

CT is the modality of choice in the postoperative setting, being able to detect and differentiate, even in the earliest phases, between normal and pathological findings. Other imaging techniques, like MRI with MR-cholangiopancreatogaphy (MRCP) sequences and fluoroscopy, are less useful and are used mainly for few specific indications [4].

In this article we will review the main types of major pancreatic resections and the resulting postoperative anatomy, normal findings in the early postoperative time and main post-surgical complications.

## Surgical background

Different surgical procedures are performed based on the type of lesion and its location and can be broadly divided into resection and drainage procedures [2]. The latter, however, will not be discussed in this review.

The most commonly performed resections are pancreaticoduodenectomy (pancreaticoduodenectomy) and distal pancreatectomy (distal pancreatectomy). Pancreaticoduodenectomy is performed for diseases involving the head of the pancreas, most commonly periampullary neoplasms, pancreatic head trauma and chronic pancreatitis [2, 6, 9].

**Fig. 1:**
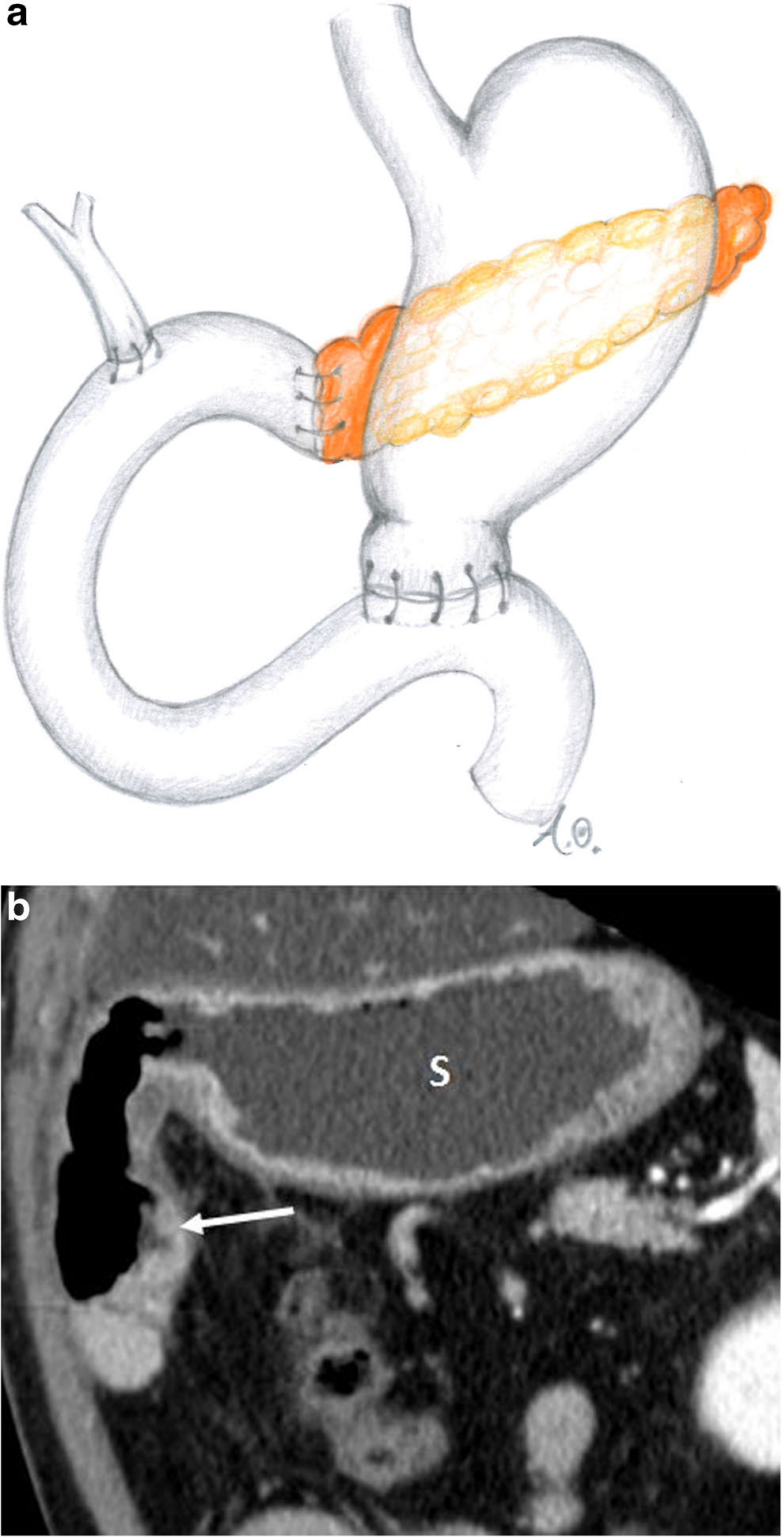
Whipple procedure (**a**, **b**). **a** Drawing. **b** Coronal CT image. The stomach (s) and the gastrojejunostomy (white arrow) after the Whipple procedure are visible

**Fig. 2:**
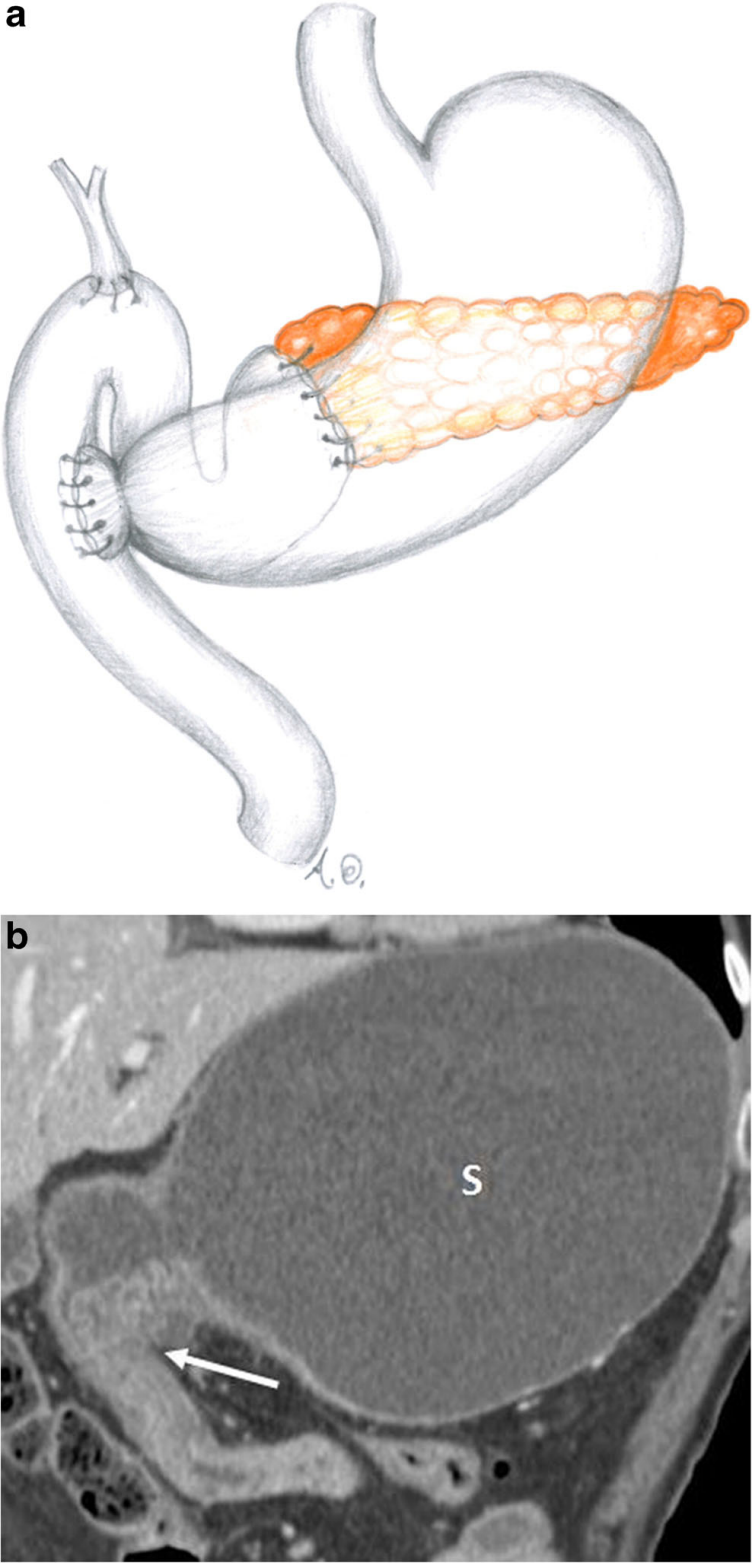
Pylorus-preserving pancreaticoduodenectomy (**a**, **b**). **a** Drawing. **b** Coronal CT image. The duodenojejunostomy (white arrow) is visible. The stomach (s) is visualised

There are two different variants of pancreaticoduodenectomy: the Whipple and pylorus-preserving procedures. Both include resection of the pancreatic head, duodenum, gallbladder, distal bile duct, proximal jejunum and regional lymph nodes with the creation of a hepaticojejunostomy and a pancreaticojejunostomy. In the Whipple procedure, the gastric antrum is removed with the creation of a gastrojejunostomy (Fig. 1), whereas the pylorus-preserving variant retains the gastric antrum and the first portion of the duodenum with the creation of a duodenojejunostomy (Fig. 2). The pylorus-preserving procedure reduces surgical time and intraoperative bleeding. It was originally introduced with the goal of improving gastric motility and reducing the incidence of marginal ulcers and alkaline gastritis. This, however, was not achieved in practice [10].

In both types of pancreaticoduodenectomy the pancreatic remnant can be anastomosed to the stomach, thus creating a pancreatico-gastro anastomosis instead of a pancreaticojejunostomy. These two types of pancreatic anastomosis seem to have the same rate of complications, although a recent meta-analysis revealed that pancreatico-gastro anastomoses have a lower rate of pancreatic fistula [11].

**Fig. 3:**
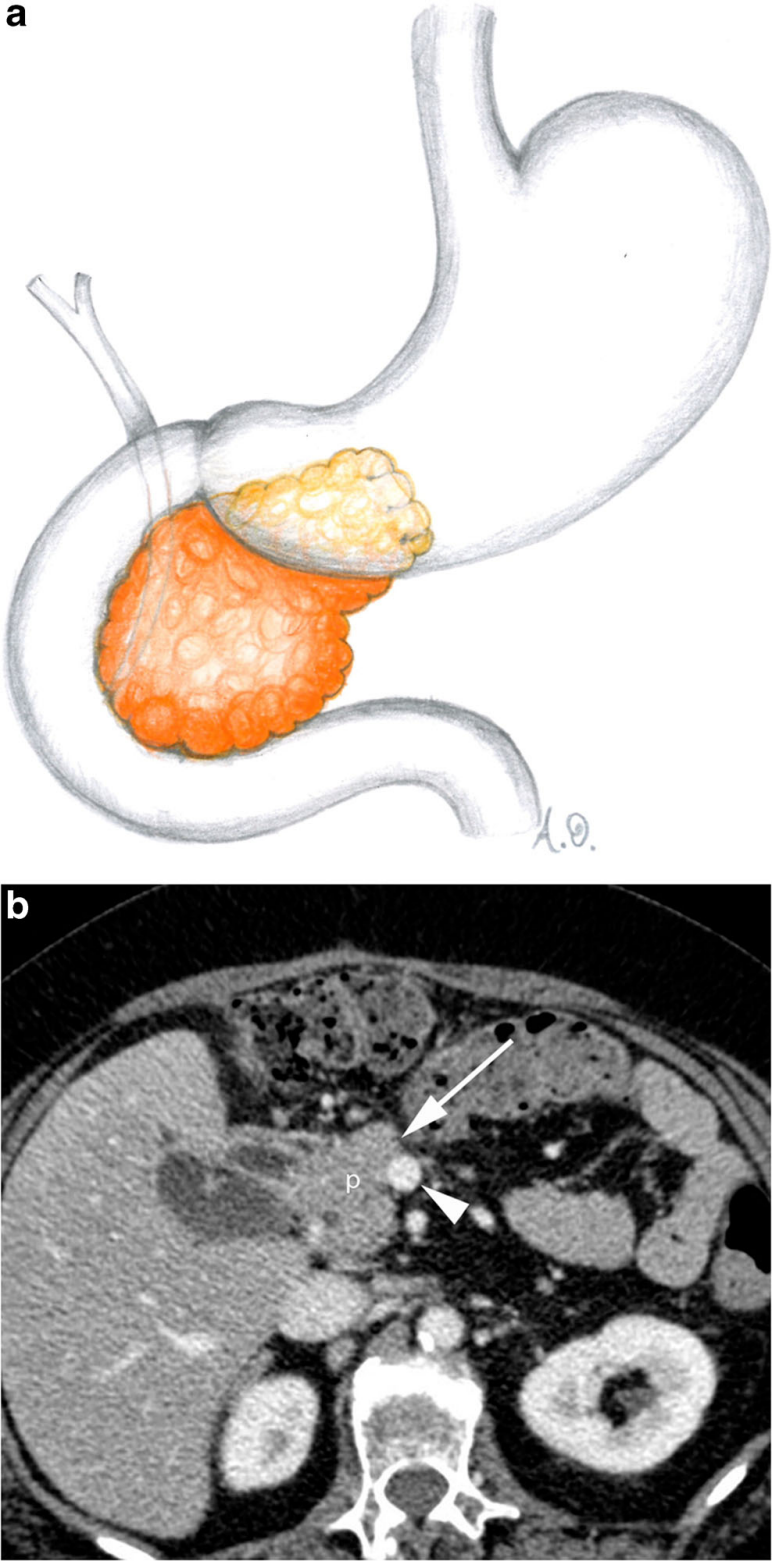
Distal pancreatectomy (**a**, **b**). **a** Drawing. **b** Axial CT image. The head of the pancreas (p) is visible after a distal pancreatectomy. The resection margin (white arrow) is located at the level of the superior mesenteric vein (white arrowhead)

Distal pancreatectomy is performed for lesions located in the body or tail of the pancreas: the distal portion of the pancreas is resected at or to the left of the superior mesenteric vein (Fig. 3). Usually distal pancreatectomy is associated with splenectomy. In this procedure, no anastomoses are created and the post-surgical anatomy is almost normal [2–4, 9].

**Fig. 4:**
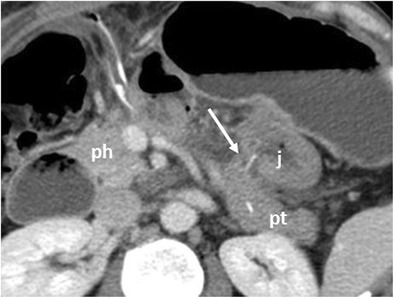
Central pancreatectomy. The head of the pancreas (ph) and the pancreatic tail (pt) are visible. The pancreaticojejunostomy to the distal pancreatic remnant (white arrow) and the anastomotic jejuna loop (j) are visible

Central pancreatectomy is a rarely performed procedure, used in case of benign lesions or low malignant neoplasms, e.g., G1 neuroendocrine tumour (NET) and intraductal papillary mucinous neoplasia (IPMN). In this procedure, only a portion of the body of the pancreas is resected to spare the pancreatic parenchyma to preserve both endocrine and exocrine functions. The reconstructive time in this procedure requires a Roux-en-Y pancreaticojejunostomy or a pancreaticogastrostomy to the distal pancreatic remnant (Fig. 4) [2, 3, 9, 12, 13].

## Imaging modalities

In the first postoperative period no imaging is required unless complications are suspected.

As stated above, CT is the modality of choice to evaluate the postoperative patient, because it is widely available, fast and allows exploring the entire abdomen, with high spatial and contrast resolution. For these reasons it is able to clearly define the postoperative anatomy allowing identification of the anastomoses. It is also able to demonstrate para-physiological postoperative changes and early and late true complications such as pancreatic fistula, haemorrhage, acute pancreatitis of the remnant, abscess, aneurysms, biliary anastomotic stenosis and local recurrence [4, 6].

MR has similar performance to CT in postoperative conditions, but it is more expensive, time consuming, less available and requires greater compliance from the patient. As a consequence, MR with MRCP sequences is mainly performed to study the biliary and pancreatic ductal systems and anastomoses.

Other imaging modalities, such as fluoroscopy, can provide information in relation to specific questions such as the evaluation of gastrointestinal function or of the hepatico- and pancreatico-anastomosis.

To evaluate resected patients in the first postoperative period, we use a multiphase technique including a non-contrast scan, useful to recognise hyperdense materials (clips, stents or blood), followed by a late arterial phase (bolus tracking, 200 HU threshold, 15 s delay) and a venous phase (60 s delay after the threshold has been reached). Patients receive 1.5 ml/kg of high-concentration nonionic contrast material, at a rate of 3–4 ml/s, followed by a 50-ml saline bolus.

The MRI acquisition protocol in the postoperative setting at our institution is based on multiplanar T1- and T2-weighted sequences with and without fat saturation, diffusion-weighted images and 3D MRCP acquisitions. Multiphase acquisitions after the administration of hepatospecific contrast agent are performed; when clinically indicated, a late scan in the excretory phase is performed to evaluate the biliary system.

These protocols allow studying the pancreatic parenchyma and the entire abdomen to identify all possible complications.

## Normal postoperative findings

**Fig. 5:**
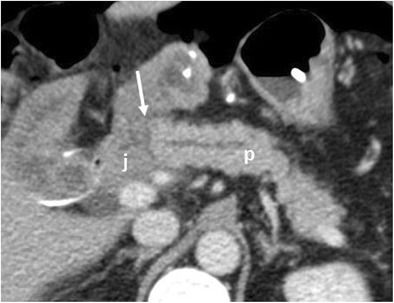
Pancreaticojejunostomy. The pancreas (p), jejunal anastomotic loop (j) and pancreaticojejunostomy (white arrow) are visible after a Whipple procedure

When evaluating a postoperative CT, depending on the type of surgery, the first assessment should be of the anastomoses [2, 4, 6, 14, 15]:

1. Pancreaticojejunostomy: a jejunal loop is anastomosed to the right of the pancreatic remnant, anteriorly to the superior mesenteric artery (Fig. 5).
2. Pancreaticogastrostomy: the pancreas remnant is anastomosed with the posterior wall of the stomach (Fig. . 6).
3. Hepatico-jejunostomy: the jejunal loop is located at the hepatic hilum (Fig. 7a). This anastomosis can be well evaluated by MR with MRCP sequences (Fig. 7b).
4. Gastro-/duodenojejunostomy: gastrojejunostomy usually is located anteriorly and on the right of the pancreatic remnant. Duodenojejunostomy is usually located in the right upper quadrant of the abdomen (Fig. 1).

**Fig. 6:**
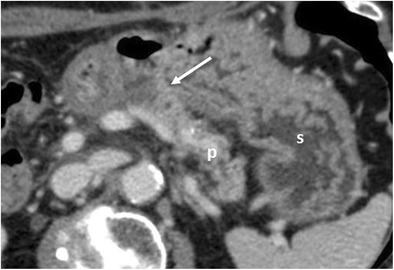
Pancreaticogastrostomy. The pancreas (p), stomach (s) and pancreaticogastrostomy (white arrow) are visible after a pancreaticoduodenectomy

**Fig. 7:**
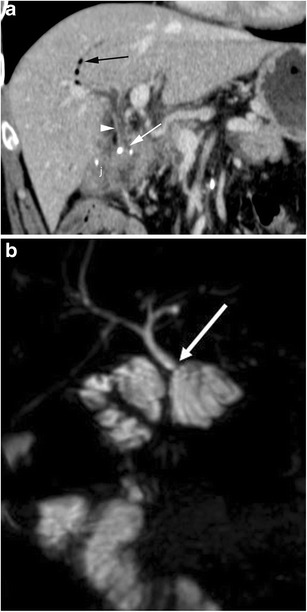
Hepaticojejunostomy (**a**, **b**). **a** The hepaticojejunostomy (white arrow) and jejunum (j) are visible. The common hepatic duct (white arrowhead) is slightly dilated and a small amount of aerobilia (black arrow) is visualised. **b** MRCP better depicts the hepaticojejunostomy (white arrow)

In the first postoperative period CT may show a series of findings that should be regarded as “normal” physiological temporary consequences of surgery. Among these, the most common are pneumobilia, perivascular cuffing, fluid collections, lymphadenopathies, acute anastomotic oedema, peripancreatic fat stranding and presence of stents and free air [2, 4, 6, 16].

### Pneumobilia-pneumowirsung

**Fig. 8:**
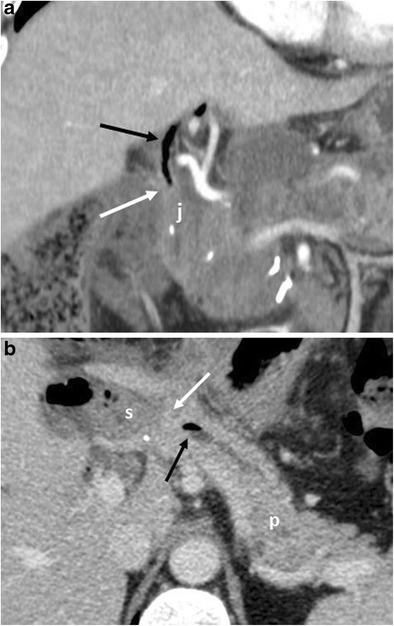
Pneumobilia and pneumowirsung (**a**, **b**). **a** There is a fair amount of air in the main and in left bile ducts (black arrow). The hepaticojejunostomy (white arrow) and anastomotic jejunal loop (j) are visualised. **b** The pancreas (p), anastomotic jejunal loop (s) and pancreaticojejunostomy (white arrow) are visualised. The main pancreatic duct is mildly dilated with an air bubble within (black arrow)

Air can be seen in both the biliary tract and lumen of the main pancreatic duct. Pneumobilia is far more common (67–80% of cases) and typically is much more evident in the left biliary tree (Fig. 8a). The presence of air can be exploited to identify the pancreatic or biliary anastomosis (Fig. 8b) [3, 16].

### Perivascular cuffing

**Fig. 9:**
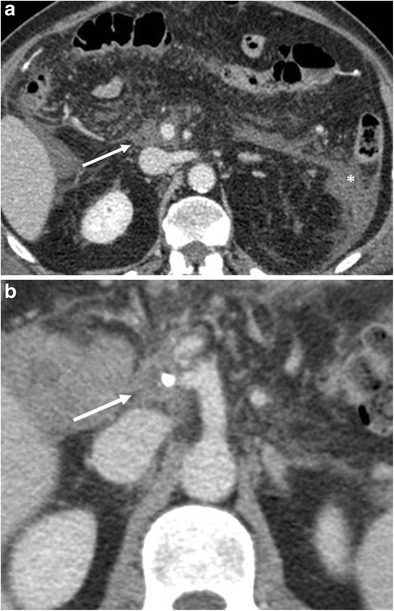
Perivascular cuffing. **a**, **b**) Axial CT images show a thickening (white arrows in **a** and **b**) of the fat tissue surrounding the superior mesenteric vessels in **a** and caeliac trunk in **b**. This stranding can be very focal and mass-like. In **a**, a postoperative reactive thickening of the anterior left pararenal fascia and a fluid collection on the same side (*) are visible

Perivascular cuffing is a soft-tissue stranding in the mesenteric fat that can occur within the surgical bed and surrounding the caeliac axis and its branches and the superior mesenteric artery. This cuffing is due to an inflammatory reaction and can be observed in up to 60% of patients. It can potentially be extremely focal and mass-like in appearance (Fig. 9). However, in patients with negative surgical margins, in the first postoperative period this finding should not be mistaken for residual disease or local recurrence [2, 4, 6, 16].

### Fluid collections

**Fig. 10:**
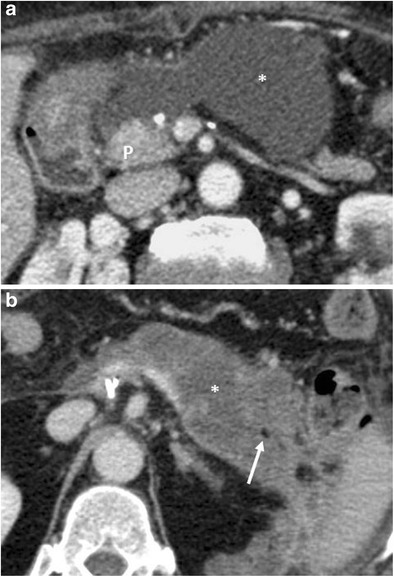
Postoperative collections (**a**, **b**). **a** Axial CT image shows a homogeneous fluid collection with a thin wall at the level of the resection margin and in the surgical bed (*) after a distal pancreatectomy. The head of the pancreas (p) is visualised. **b** An inhomogeneous necrotic fluid collection (*) with fat globules inside (white arrow) is visible within the left anterior pararenal fascia. These findings are consistent with a necrotic collection with steatonecrosis due to a postoperative pancreatitis after a Whipple procedure

In the early postoperative period (first 14 days), thin-walled or poorly delineated fluid collections are seen in about 28.5% of the cases, usually in the surgical bed and near the anastomoses (Fig. 10 a) [2, 4, 16]. These collections at both CT and MRI are homogeneous with pure fluid attenuation or homogeneous signal intensity on both T1- and T2-weighted sequences. These fluid collections are transient and should regress in the next 3–6 months and do not require any treatment [6]. Attention should be paid to the presence of blood products, which will appear relatively hyperdense on non-contrast-enhanced CT images. A more irregular texture should raise the suspicion of superinfection or steatonecrosis (Fig. 10b). The presence of air bubbles within fluid collections is another important feature that can be associated with infection or fistula. Significant fluid collections associated with pancreatic fistula or abscesses are usually treated with image-guided percutaneous drainage in the presence of serious clinical symptoms such as fever, pain or sepsis [7].

### Lymphadenopathy

**Fig. 11:**
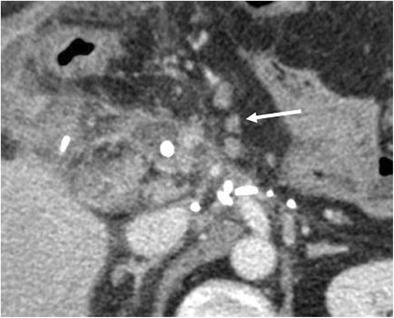
Inflammatory adenopathy. Axial CT image shows the presence of enlarged lymph nodes (white arrow) in the mesentery, close to the surgical bed

Enlarged lymph nodes are a common finding following pancreatic resection. They are more commonly identified surrounding the surgical bed and in the mesentery (Fig. 11). These are almost always reactive lymphadenopathies, although they may be quite large, with a short axis greater than 1 cm, and should regress within 6 months at follow-up imaging [6, 15].

### Acute anastomotic oedema

**Fig. 12:**
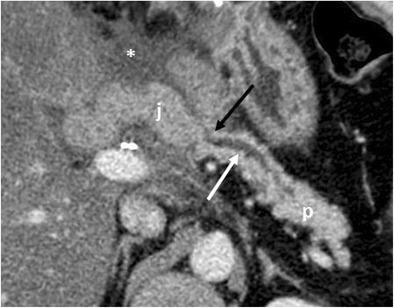
Oedema of the pancreaticojejunostomy. Axial CT image in the immediate postoperative period shows a mild thickening of the jejunum (j) at the pancreatic anastomosis (black arrow) due to acute oedema. This condition leads to a dilation of the main pancreatic duct (white arrow). A fluid collection is visible surrounding the anastomotic jejunal loop (*)

A thickening of the pancreatic anastomosis is a common finding and is due to acute postoperative oedema. This could lead to a dilation of the main pancreatic duct. Oedema of the biliary anastomosis can cause mild intrahepatic biliary dilation. These early findings should not be misinterpreted as anastomotic stricture and will typically improve with time (Fig. 12) [2, 6].

### Stranding of the peripancreatic fat

**Fig. 13:**
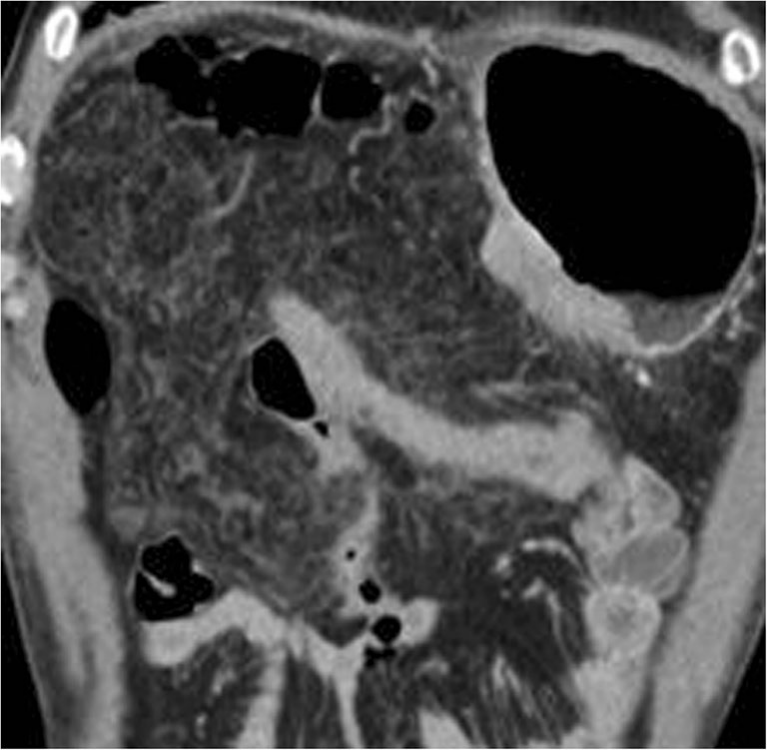
Fat stranding. Coronal CT image in the immediate postoperative period shows a diffuse stranding of the mesenteric fat tissue

Fat stranding is a very common finding (29–50% of cases) and does not represent a diagnostic challenge [3]. It is most commonly visible in the peripancreatic fat or at the root of the mesentery. The involvement of visceral fat can be diffuse, with diffuse alterations involving the mesentery or the retroperitoneal fat (Fig. 13). Stranding appears as an ill-defined increased attenuation of the fat tissue with a linear or reticular appearance [17]. It is often associated with perivascular cuffing [16] and will resolve spontaneously within 3–6 months after surgery [6].

## Complications

### Postoperative pancreatic fistula

Pancreatic fistula is the most common complication after partial pancreatic resection, occurring between 10% and 30% of cases, and it is associated with increased length of hospital stay, costs and mortality [7, 18–20]. According to the ISGPF (International Study Group of Pancreatic Fistula) definition, pancreatic fistula is “the presence of drainage fluid on the third postoperative day or later, with an amylase content greater than three times the upper normal serum value” [21]. Pancreatic fistula represents the failure of healing/sealing of the pancreatic anastomosis in pancreaticoduodenectomy or a parenchymal leak from the raw resection margin in distal pancreatectomy [7, 18, 22]. The major risk factors are a small-calibre main pancreatic duct (diameter < 3 mm), a soft parenchymal texture and intraoperative bleeding [6, 7]. Imaging studies, especially CT, can confirm the clinical suspicion of pancreatic fistula. Imaging acquisition should be guided by clinical signs and symptoms and laboratory data as the diagnostic criteria mentioned above are fulfilled.

**Fig. 14:**
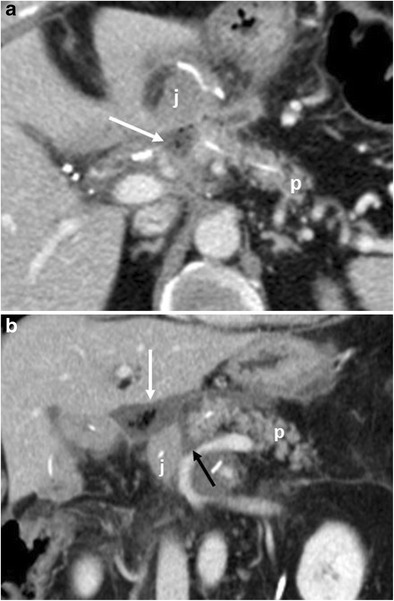
Pancreatic fistula. **a**, **b** Multiplanar CT images. The pancreatic stump (p) and jejunal loop (j) are visualised. A complete disruption of the pancreatic anastomosis is evident (black arrow in **b**). At the level of the anastomosis a fluid collection with multiple air bubbles inside is visible (white arrows), a finding that strongly suggests the presence of a pancreatic fistula

CT features suggestive of the presence of pancreatic fistula in pancreaticoduodenectomy include: fluid collections around the pancreaticojejunostomy site or in the pancreatic bed, air bubbles in a peripancreatic collection and disruption of the pancreatic anastomosis (Fig. 14) [6, 7].

**Fig. 15:**
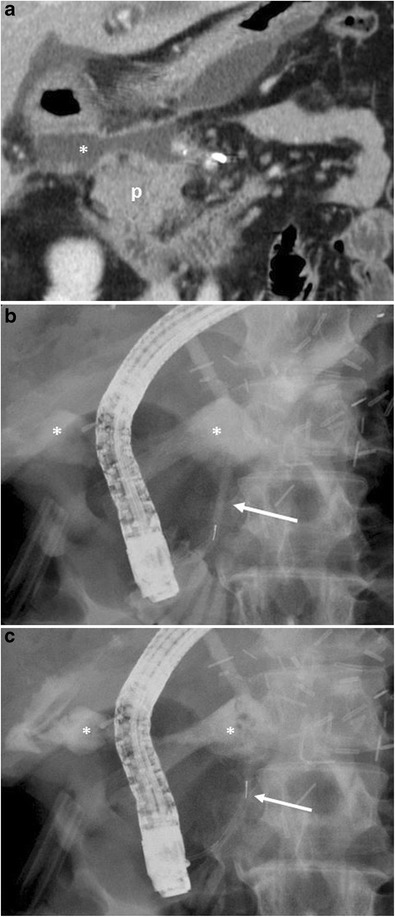
Pancreatic fistula. **a** Coronal curvilinear CT reconstruction shows the presence of a fluid collection (*) close to the resection margin of the pancreas (p) after a distal pancreatectomy. The presence of amylase from a surgical drainage (not shown in **a**) was consistent with a pancreatic fistula. **b**, **c** Spot images during ERCP show the passage of contrast material through the main pancreatic duct (white arrows) in the collection (*), a finding diagnostic for a leakage of pancreatic juice at the resection margin

The CT appearance of pancreatic fistula arising after a distal pancreatectomy consists of a collection at the level of the resection margin with or without an associated fistulous tract (Fig. 15).

**Fig. 16:**
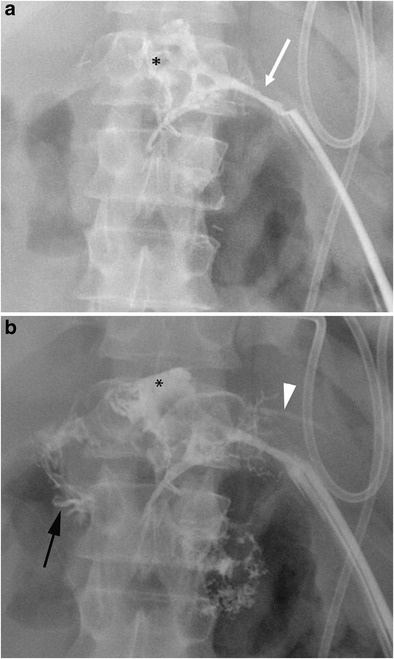
Pancreatic fistula after the Whipple procedure. **a**, **b** Sequential images acquired during fistulography. Contrast medium is injected through a drainage on the left. Immediate filling of a fistulous tract (white arrow) and a collection (*) is seen. In the later phases passage of contrast medium inside the anastomotic loop becomes evident (black arrow in **b**), findings diagnostic for an anastomotic dehiscence. The main pancreatic duct is visualised (white arrowhead in **b**)

When CT does not demonstrate the typical findings of a pancreatic fistula, conventional fistulography can confirm the dehiscence of the anastomosis. In this dynamic examination, iodinated contrast is injected under fluoroscopy control through the drainage located closer to the anastomosis: the passage of the injected contrast into the enteric lumen is diagnostic of pancreatic fistula (Fig. 16) [7, 22]. After distal pancreatectomy endoscopic retrograde cholangiopancreatography (ERCP) can demonstrate the presence of pancreatic fistula although this procedure is never used as diagnostic tool. Thus, the demonstration of the leakage is usually found as an ancillary finding in patients who undergo ERCP for other reasons (e.g., lesions of the biliary tract).

### Delayed gastric emptying (DGE)

**Fig. 17:**
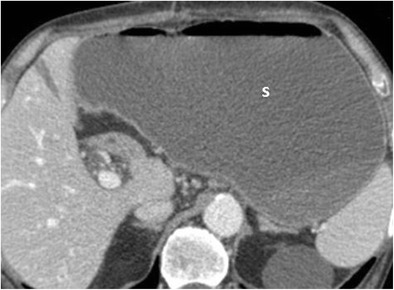
Axial CT image. A severely distended stomach filled with fluid and air is evident (s)

Delayed gastric emptying is the second most common complication after pancreaticoduodenectomy, with a reported prevalence between 20% and 50% [8]. It has been defined by the ISGPS (International Study Group of Pancreatic Surgery) as “the impossibility of resuming oral feeding after the first postoperative week or the prolonged use of a nasogastric aspiration tube” [8, 23]. Recent meta-analyses and prospective studies revealed that there are not significant differences regarding surgical technique, in particular whether the pylorus is preserved or not [24, 25]. Risk factors reported in the literature include cholangitis, diabetes and prior abdominal surgery [8]. Although the diagnosis is not based on imaging but on clinical symptoms, the presence of a severely distended stomach at CT is highly suggestive (Fig. 17).

### Postoperative bleeding

Bleeding is a quite common complication, occurring in 2% to 16% of cases after pancreatic resection, and it is burdened by a high mortality (38%) [8, 26, 27].

**Fig. 18:**
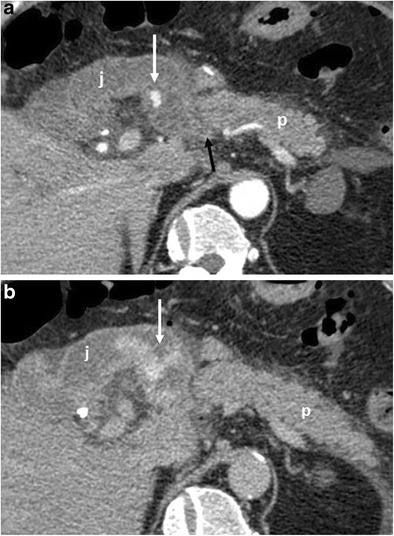
**a**, **b** Axial CT curvilinear reconstructions show an active extravasation in the arterial phase (white arrow in **a**) within the lumen of the jejunal anastomotic loop (j). Bleeding becomes more evident in the late phase (white arrow in **b**). The pancreatic stump is seen (p)

**Fig. 19:**
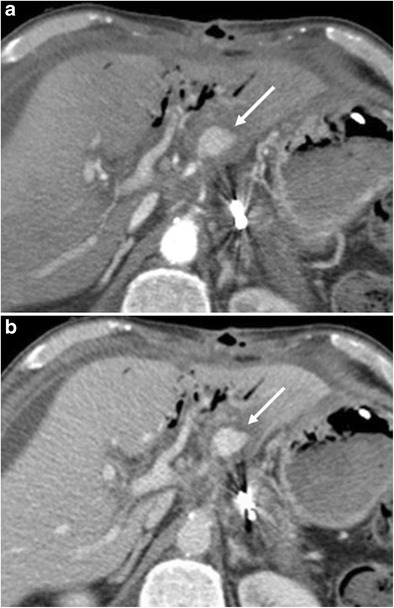
**a**, **b**) Axial CT images show an active extravasation in the arterial phase (white arrow in **a**) coming from the common hepatic artery after a Whipple procedure. Bleeding becomes more evident in the venous phase (white arrow in **b**)

Bleeding can be classified into early (< 24 h) or late (> 24 h) on the basis of the interval after surgery. They can also be divided into intra- or extra-luminal based on their location [28]. Intra-luminal haemorrhage presents with haematemesis or melaena (Fig. 18). Extra-luminal bleeding is more common and presents with blood in the drainage fluid (Fig. 19) [8]. In most cases haemorrhage results from active bleeding of the gastroduodenal artery stump and could be related to inadequate surgical ligation, vascular erosion or pseudoaneurysm formation (usually secondary to pancreatic fistula). In haemodynamically stable patients, multiphase CT is the imaging technique of choice because of its high temporal and spatial resolution. Unenhanced CT can identify the presence of haematomas or blood in the gastro-intestinal lumen. After the injection of contrast it is possible to identify sites of active extravasation or pseudoaneurysm formation [6]. The chance to use dual-energy computer tomography (DECT) to identify haematomas using virtual non-contrast acquisition to reduce radiation exposure is well known. Nonetheless, up to now, in the clinical setting of acute postoperative bleeding, DECT is not suggested as a useful imaging investigation [29].

### Hepatic infarction

Hepatic infarction is a rare event, with a prevalence of 1% [8, 30]. Infarction is uncommon because of the dual hepatic blood supply from the hepatic artery and portal vein and it can be due to an insult to the hepatic artery or portal vein. However, the presence of a pre-existing severe stenosis of the superior mesenteric artery or the caeliac trunk is a significant risk factor that can lead to infarction [7] even without any intraoperative arterial trauma or venous impairment [6, 31].The imaging appearance of an hepatic infarction could be due to ischaemia of the biliary tree that has an almost exclusive arterial blood supply, thus being more sensitive to arterial lesions during surgery [32]. The left hepatic lobe is more commonly affected following a trauma during the dissection of the hepatic artery and the caeliac trunk. The inadvertent sacrifice of a right hepatic artery arising from the superior mesenteric artery (replaced right hepatic artery) may lead to a selective infarction of the right lobe [6, 33].

**Fig. 20:**
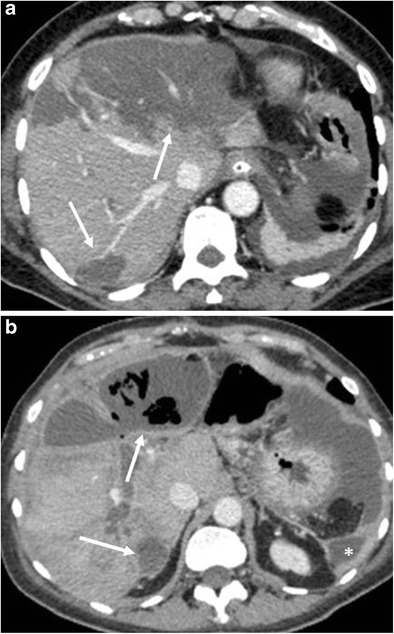
Hepatic infarction. **a** CT image shows multiple hypodense and hypovascular areas of infarction (white arrows) following a pancreaticoduodenectomy. **b** CT image 4 weeks later in the same patient. The areas of infarction show a thickened and enhancing wall with multiple air bubbles within (white arrows), findings consistent with hepatic abscesses. Another abscess is visible at the level of the left lateroconal fascia (*)

Ischaemic lesions at CT appear as hypodense and hypovascular areas with sharp margins, without mass effect (Fig. 20). At MRI these areas are hypointense on T1- and hyperintense on T2-weighted images, hypoenhancing after contrast administration.

### Postoperative pancreatitis

**Fig. 21:**
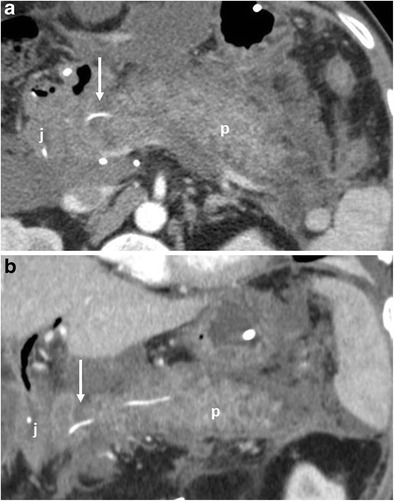
Acute pancreatitis. **a**, **b** The pancreatic remnant (p), jejunal loop (j) and pancreatic anastomosis (white arrow) are visible. The pancreas is thickened and oedematous, surrounded by a discrete amount of fluid that extends along the anterior pararenal fascia, findings highly suggestive of an acute necrotic postoperative pancreatitis

The incidence of pancreatitis is 2–3% [6, 8]. Fat stranding and inflammatory changes in the surgical bed and surrounding the pancreatic remnant are common findings, making the differentiation between pancreatitis and normal postoperative inflammation challenging, especially in cases of mild pancreatitis. Moreover, the serum levels of amylase and lipase are unreliable in the postoperative period [8, 34]. Imaging should be performed when there is a clinical suspicion of pancreatitis. In severe cases CT can make the diagnosis showing severe peripancreatic inflammatory changes, fluid and infiltration. An abnormal thickening of the anterior pararenal fascia is suggestive for postoperative pancreatitis (Fig. 21) [6].

### Portal vein and superior mesenteric vein thrombosis (SMV)

**Fig. 22:**
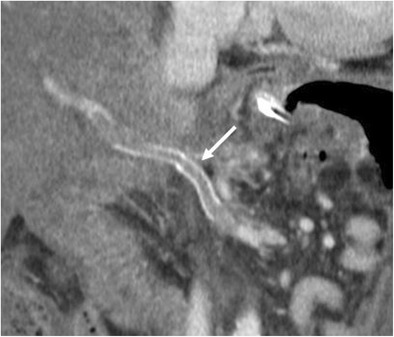
Portal vein thrombosis. CT curvilinear coronal reconstruction image shows the presence of a massive thrombosis of the superior mesenteric and portal venous axis (white arrow) following a DCP with a resection of the portal vein and prosthetic reconstruction

The development of surgical techniques and generally a more aggressive surgical approach has increased the complexity of procedures. Nowadays, regarding venous infiltration, it is possible to perform quite long segment resections thanks to the better reconstruction techniques developed over the past decades. Accordingly, there has been an increase in the incidence of venous thrombosis (SMV or portal vein) after pancreatic resection, being reported in up to almost 17% [8, 35]. The development of venous thrombosis can lead to very severe consequences, potentially causing intestinal ischaemia, ascites, hepatic ischaemia and ultimately death [6]. CT is the best imaging modality to identify thrombosis due to its high spatial resolution and multiplanar reconstruction, especially in the coronal plane. The typical CT feature is a filling defect within the lumen of the SMV or portal vein, better depicted in the portal venous phase [6, 8]. At MRI the thrombus has different pre-contrast signal intensities depending on the time of onset, being hyperintense in both T1- and T2-weighted images in the acute setting and hypointense in T2 in the chronic phase. After contrast administration a filling defect may be visible (Fig. 22) [36].

### Abscesses

**Fig. 23:**
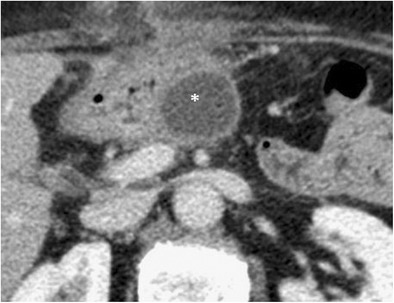
Abdominal abscess. Axial CT image shows the presence of a fluid collection with a thick and enhancing wall (*) close to posterior aspect of the stomach

The overall incidence of abscesses ranges up to 6% [6, 8, 16]. The presence of an abscess may be suspected as the patient develops fever or a septic status. Usually they arise because of superinfection by enteric bacteria of a pre-existing acute postoperative fluid collection especially when associated with a leakage from one of the anastomoses [6, 8, 37]. Abscesses may also develop separately from collections because of a contamination during the surgical procedure or the colonisation of the surgical drainage [38]. In the end, an abscess may also result from superinfection of an ischaemic hepatic area [6, 8]. CT shows the presence of a fluid collection with a thickened and enhancing wall (Fig. 23). The presence of air within a collection or necrotic hepatic parenchyma is strongly suggestive for the diagnosis (Fig. 20b) [2, 4, 6, 16, 39].

### Biliary anastomotic leaks

**Fig. 24:**
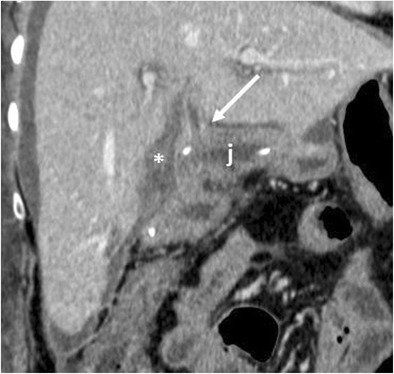
Biliary fistula. Coronal oblique CT image shows the biliary anastomosis (white arrow) surrounded by an ill-defined fluid collection (*). The jejunal loop is visible (j)

**Fig. 25:**
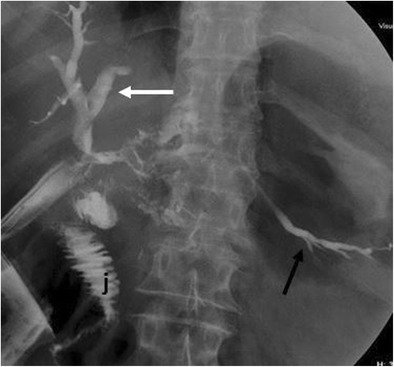
Spot image obtained during fistulography shows the passage of contrast medium through the biliary anastomosis inside the jejunal loop (j), a finding diagnostic for an anastomotic dehiscence. The biliary tree (white arrow) and the main pancreatic duct (black arrow) are also visible

Bile leaks are relatively rare, reported in 1 to 5% of cases, and usually related to a technical failure [20, 40]. The diagnosis is based on clinical and laboratory data, being defined as “a bilirubin concentration in the drain fluid at least three times the serum bilirubin concentration on or after postoperative day 3, or as the need for radiologic or operative intervention resulting from biliary collections or bile peritonitis” [8, 41]. CT can show a fluid collection near the biliary-enteric anastomosis (Fig. 24). Given the proximity of the pancreatic anastomosis, a differential diagnosis between bile leak and pancreatic fistula based only on imaging findings is almost impossible. The presence of bile in the surgical drainage is an important finding to suggest the right diagnosis. Fistulography from the surgical drainage closer to the anastomosis can highlight the passage of contrast medium into the jejunal loop and the biliary tree through the biliary anastomosis, thus confirming the diagnosis (Fig. 25). MR can reveal the presence of a fluid collection and, with the use of hepatospecific contrast agents, also confirm the bile leak in the late phases [42–44].

### Anastomotic stricture

**Fig. 26:**
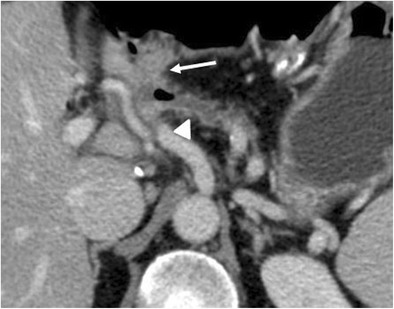
Anastomotic stricture. Axial CT image shows a dilation of the main pancreatic duct associated with atrophy of the pancreatic parenchyma (white arrowhead). No signs of local tumour recurrence are seen at the pancreatic anastomosis (white arrow)

**Fig. 27:**
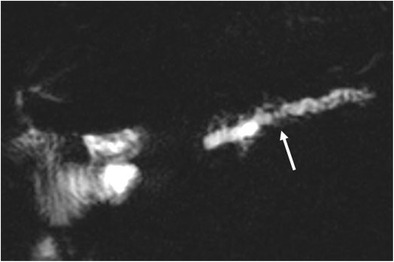
MRCP reveals a marked dilation of the main pancreatic duct and of some branch ducts (white arrow)

Anastomotic stricture is the most common delayed complication after pancreaticoduodenectomy and can be identified at both the pancreaticojejunostomy (4.6% at 5 years) and hepaticojejunostomy (8.2% at 5 years) sites [6]. Ultrasound, CT and MRI can all demonstrate a dilation of the biliary tree or the main pancreatic duct. CT and MRI, along with the dilation of the main pancreatic duct, are able to highlight the consequent progressive atrophy of the pancreatic parenchyma (Fig. 26). Overall, MRI, thanks to its high contrast resolution and the chance to perform MRCP sequences, is the best imaging technique to evaluate the ductal systems and the calibre of the anastomoses (Fig. 27) [44]. The presence of a dilation of the biliary system and/or pancreatic duct may be due to progressive fibrosis at the site of the anastomosis, but an extremely careful evaluation has to be made to identify any signs of local tumour recurrence resulting in ductal obstruction [3, 4, 6, 44].

### Tumour recurrence

**Fig. 28:**
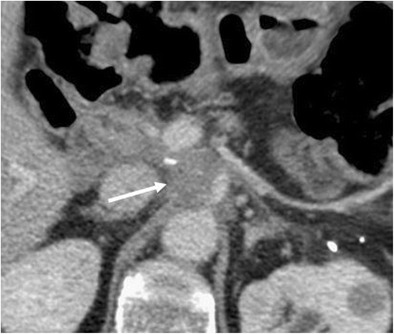
Tumour recurrence. Axial CT image shows the presence of hypodense soft tissue (white arrow) consistent with tumour recurrence, encasing the origin of the caeliac trunk and portal vein

**Fig. 29:**
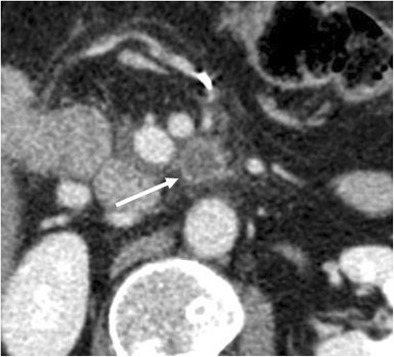
Nodal tumour recurrence. Axial CT image shows the presence of an enlarged necrotic lymph node (white arrow) along the superior mesenteric vessels, consistent with tumour recurrence

The detection of tumour recurrence in the surgical bed or as distant metastases is essential to define the prognosis of the patient and to plan any further therapies. The recurrence of pancreatic neoplasm may be local resulting in an infiltrating mass in the surgical bed or as soft tissue surrounding the mesenteric vessels (Fig. 28). The neoplastic tissue may involve the anastomoses leading to a dilation of the biliary tree or of the main pancreatic duct. The presence of loco-regional enlarged and hypodense lymph nodes at CT should raise the suspicion of a nodal recurrence (Fig. 29). Metastases are more commonly seen in the liver and less frequently in the lungs. Peritoneal carcinomatosis may be present along with ascites [3, 4, 16, 45, 46]. CT is an accurate modality in identifying disease recurrence thanks to its great spatial resolution and ability to explore the entire abdomen [4]. MRI has very similar potential compared to CT for local recurrence, coupled with higher sensitivity and specificity for liver metastases [4, 47–49]. Postoperative changes and tumour recurrence, especially in the early period, may have similar morphologic characteristics, being difficult to differentiate using CT or MRI. Abnormal PET/CT findings in the surgical bed with FDG uptake 3 months after surgery are suspicious of recurrence [50].

## Conclusions

Radiologists should be aware of surgical procedures, postoperative anatomy and normal postoperative imaging findings to better detect complications and recurrent disease. This does not only apply to those working in referral centres for pancreatic diseases or oncology, but also to general radiologists, who may encounter patients who underwent previous resections in their clinical practice. CT is the best imaging tool to depict the early and late “normal” postoperative findings and the complications, because of its speed and spatial and contrast resolution, coupled with post-processing capabilities, that increase the diagnostic confidence. MR can provide similar information, but in the early postoperative period it is less used because usually less readily available, requiring longer examination times and greater compliance from the patient, and more expensive.
